# A very rare case of osseous involvement of the dens axis in a 52-year-old HIV positive patient as part of secondary syphilis

**DOI:** 10.1007/s15010-025-02552-6

**Published:** 2025-05-19

**Authors:** K. Lange, C. Helbig, N. Eckardt, M. Baier, J. Guse, S. Hagel, A. Stallmach, B. T. Schleenvoigt

**Affiliations:** 1 Department of Internal Medicine IV, Jena University Hospital/Friedrich-Schiller-University, Jena, Germany; 2 Institute of Infectious Diseases and Infection Control, Jena University Hospital/Friedrich-Schiller-University, Jena, Germany; 3 Department for Radiology, Jena University Hospital/Friedrich-Schiller-University, Jena, Germany; 4 Institute of Medical Microbiology, Jena University Hospital/Friedrich-Schiller-University, Jena, Germany

A 52-year-old HIV-positive man, who has been on stable Integrase-based antiretroviral therapy for four years (CD4 cells absolute 505/µl, 29%; HIV-RNA < 20 copies/ml), presented to the emergency department with tonsillitis, fever, and neck pain.

Clinical examination revealed a temperature of 38·1 °C, painful neck stiffness, and a maculopapular rash predominantly on the trunk (Image 1). C-reactive protein (CRP) was moderately elevated at 50 mg/L (ULN < 5 mg/l), while the full blood count, liver, and kidney function tests were unremarkable. Lumbar puncture yielded nonspecific results (5 cells/µl, lactate 1·8 mmol/L, glucose 5·7 mmol/L, protein 350 mg/L). Serology indicated acute and active syphilis (TPHA: 1:2560, FTA IgG: 1:5120, VDRL: 1:256, IgM-Blot positive). The *Treponema pallidum*-specific antibody index did not show intrathecal synthesis. Surprisingly, the contrast-enhanced MRI showed bone marrow edema at the base of the dens axis with adjacent soft tissue swelling, including the tonsillar soft tissue (Image 2–4). The subsequent CT scan detected destruction of the endplate and a loss of trabecular structure in the C2 vertebral body (Image 5–6).

Secondary syphilis with bone involvement of the dens axis was diagnosed, and systemic treatment was started with intravenous penicillin (3 × 10 million units daily for 14 days), followed by oral doxycycline (2 × 100 mg daily for 42 days). In differential diagnosis, primary bone involvement as part of the syphilitic lesion in the pharynx was also considered.

At the 6-week follow-up, the patient was asymptomatic. A follow-up CT scan showed consolidation of the C2 vertebral body and a regression of the prevertebral soft tissue swelling (Image 7). In another follow-up examination after 12 weeks, further progressive consolidation and ventral overgrowth extending to the level of C3 were observed (Image 8). Follow-up serology after 5 weeks (TPHA: 1:1280, VDRL: 1:32) corresponded with the clinical improvement observed in the patient’s condition after effective treatment.

**Image 1:**
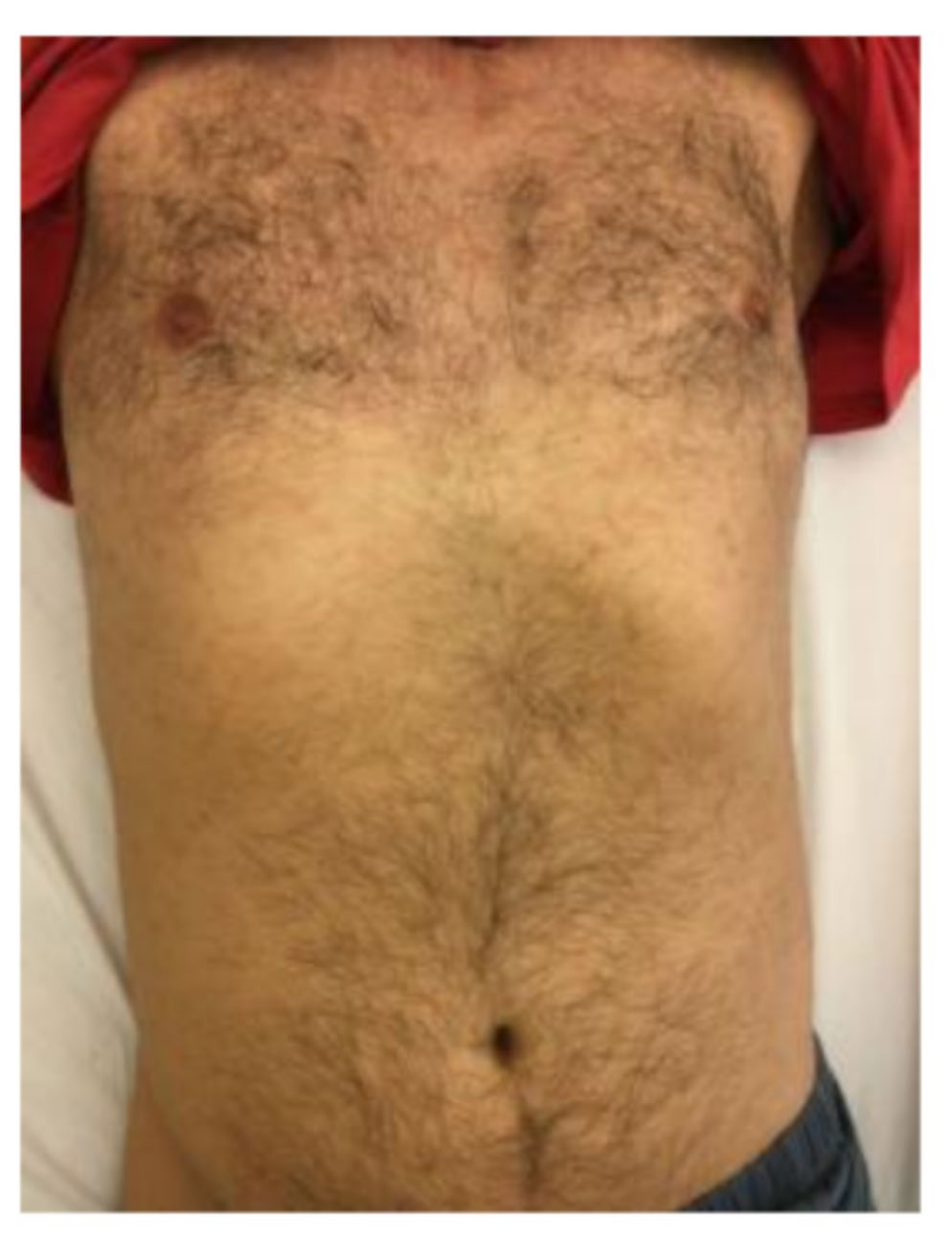
Maculopapular rash

**Image 2–4:**
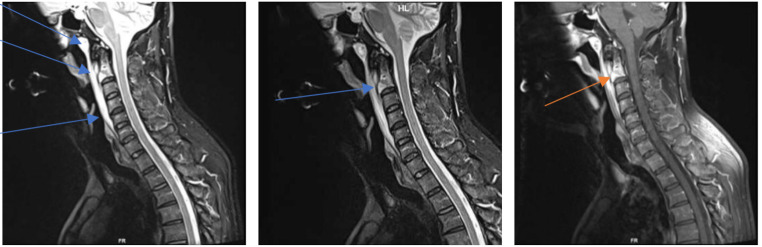
Contrast-enhanced MRI scan showing bone marrow edema of the base of the dens axis with adjacent soft tissue swelling including the soft tissue of the tonsils

**Image 5–6:**
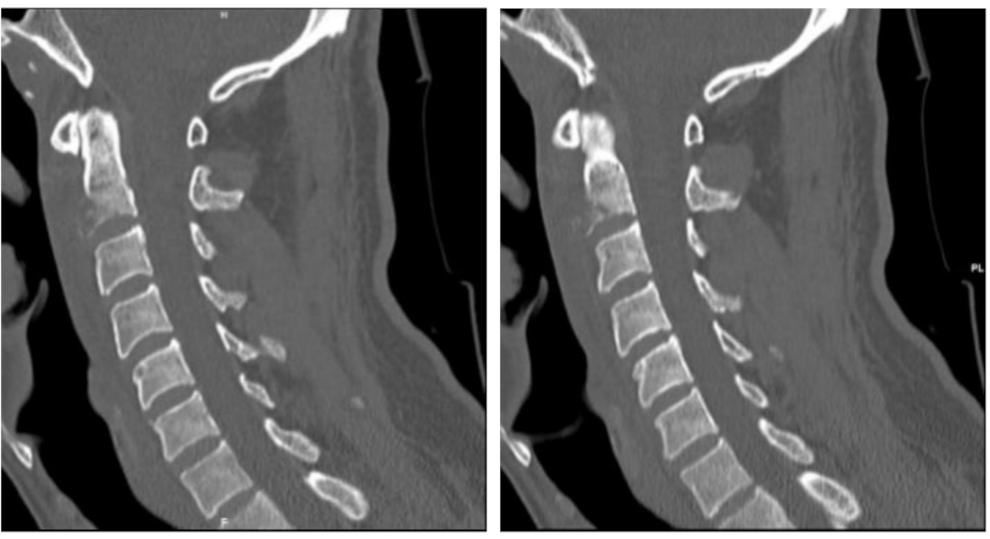
CT scan showing a destruction of the endplate and a loss of the trabecular structure in C2 vertebral body

**Image 7:**
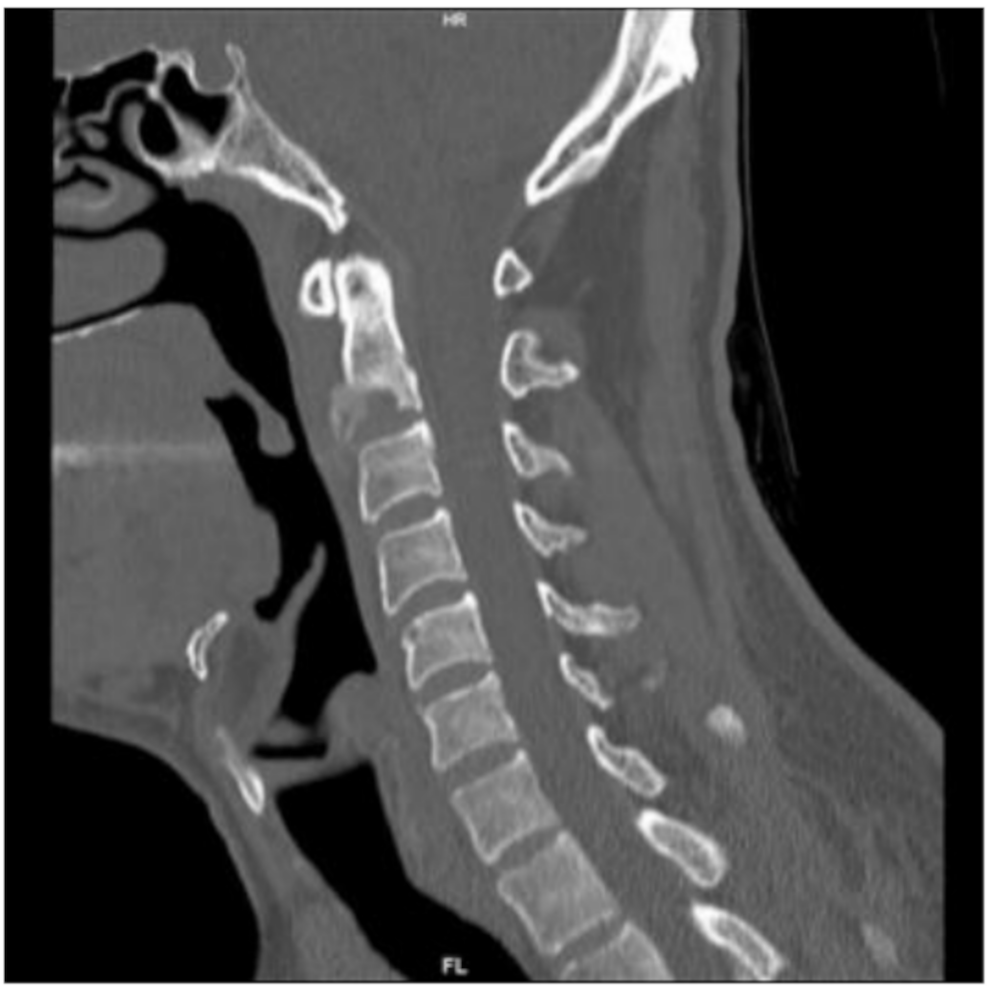
CT scan after 6 weeks showing consolidation of C2 vertebral body and a regression of the prevertebral soft tissue swelling

**Image 8:**
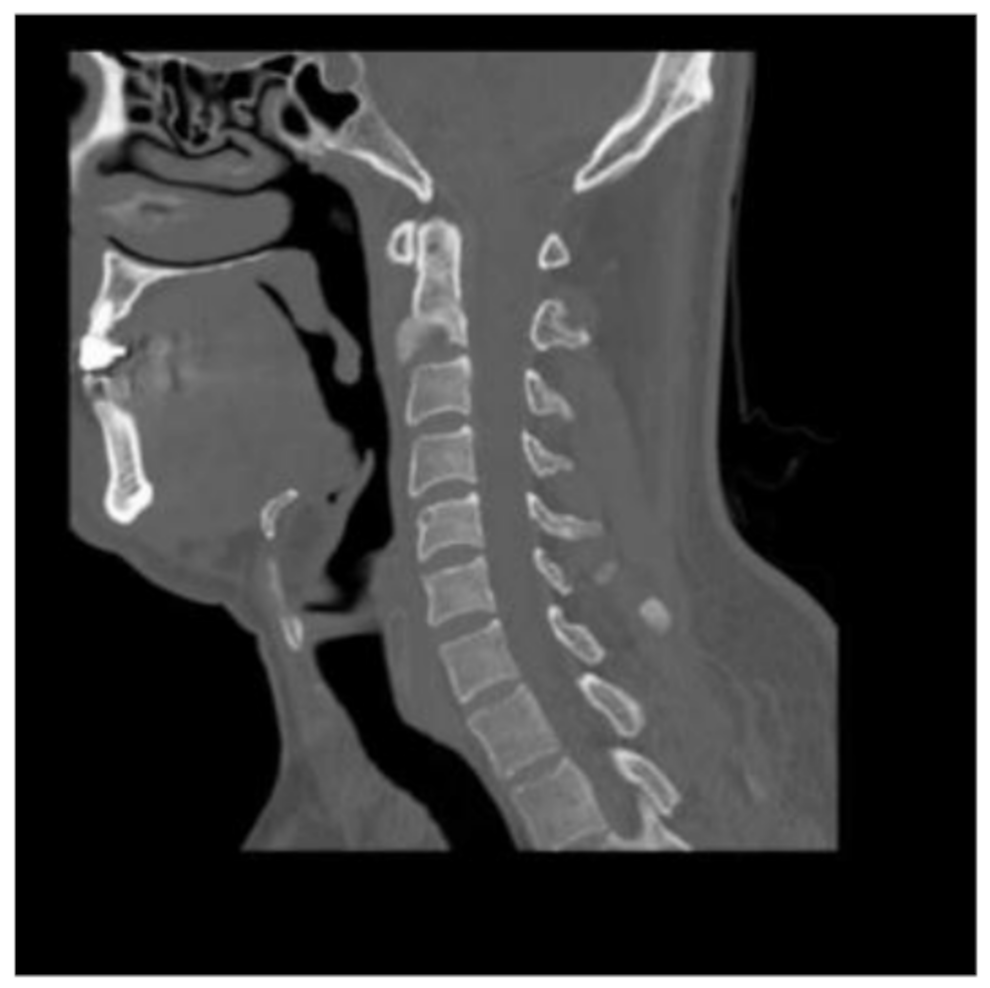
CT scan after 12 weeks showing further progressive consolidation and ventral overgrowth extending to the level of C3

## Data Availability

No datasets were generated or analysed during the current study.

